# Benchmarking Effectiveness and Efficiency of Deep Learning Models for Semantic Textual Similarity in the Clinical Domain: Validation Study

**DOI:** 10.2196/27386

**Published:** 2021-12-30

**Authors:** Qingyu Chen, Alex Rankine, Yifan Peng, Elaheh Aghaarabi, Zhiyong Lu

**Affiliations:** 1 National Center for Biotechnology Information, National Library of Medicine, National Institutes of Health Bethesda, MD United States; 2 Harvard College Cambridge, MA United States; 3 Weill Cornell Medicine New York, NY United States; 4 Towson University Towson, MD United States

**Keywords:** semantic textual similarity, deep learning, biomedical and clinical text mining, word embeddings, sentence embeddings, transformers

## Abstract

**Background:**

Semantic textual similarity (STS) measures the degree of relatedness between sentence pairs. The Open Health Natural Language Processing (OHNLP) Consortium released an expertly annotated STS data set and called for the National Natural Language Processing Clinical Challenges. This work describes our entry, an ensemble model that leverages a range of deep learning (DL) models. Our team from the National Library of Medicine obtained a Pearson correlation of 0.8967 in an official test set during 2019 National Natural Language Processing Clinical Challenges/Open Health Natural Language Processing shared task and achieved a second rank.

**Objective:**

Although our models strongly correlate with manual annotations, annotator-level correlation was only moderate (weighted Cohen *κ*=0.60). We are cautious of the potential use of DL models in production systems and argue that it is more critical to evaluate the models in-depth, especially those with extremely high correlations. In this study, we benchmark the effectiveness and efficiency of top-ranked DL models. We quantify their robustness and inference times to validate their usefulness in real-time applications.

**Methods:**

We benchmarked five DL models, which are the top-ranked systems for STS tasks: Convolutional Neural Network, BioSentVec, BioBERT, BlueBERT, and ClinicalBERT. We evaluated a random forest model as an additional baseline. For each model, we repeated the experiment 10 times, using the official training and testing sets. We reported 95% CI of the Wilcoxon rank-sum test on the average Pearson correlation (official evaluation metric) and running time. We further evaluated Spearman correlation, R², and mean squared error as additional measures.

**Results:**

Using only the official training set, all models obtained highly effective results. BioSentVec and BioBERT achieved the highest average Pearson correlations (0.8497 and 0.8481, respectively). BioSentVec also had the highest results in 3 of 4 effectiveness measures, followed by BioBERT. However, their robustness to sentence pairs of different similarity levels varies significantly. A particular observation is that BERT models made the most errors (a mean squared error of over 2.5) on highly similar sentence pairs. They cannot capture highly similar sentence pairs effectively when they have different negation terms or word orders. In addition, time efficiency is dramatically different from the effectiveness results. On average, the BERT models were approximately 20 times and 50 times slower than the Convolutional Neural Network and BioSentVec models, respectively. This results in challenges for real-time applications.

**Conclusions:**

Despite the excitement of further improving Pearson correlations in this data set, our results highlight that evaluations of the effectiveness and efficiency of STS models are critical. In future, we suggest more evaluations on the generalization capability and user-level testing of the models. We call for community efforts to create more biomedical and clinical STS data sets from different perspectives to reflect the multifaceted notion of sentence-relatedness.

## Introduction

### Background

Semantic textual similarity (STS), a measure of the degree of relatedness between sentence pairs, is an important text-mining research topic [1]. STS has been widely used in biomedical and clinical domains, including information retrieval (finding relevant sentences or passages [2]), biocuration (finding key sentences for evidence attribution [3]), and question answering (finding answer-snippet candidates [4]). Despite its importance, expertly annotated STS data sets are lacking in the biomedical and clinical domains. For example, STS-related data sets in the general domain have been developed for nearly a decade, with almost 30,000 annotated sentence pairs in total [5], whereas similar data sets in the biomedical and clinical domains had only hundreds of pairs in total before 2018 [6]. The organizers of the Open Health Natural Language Processing (OHNLP) Consortium have dedicated efforts to expanding such data sets and establishing STS open challenges in the clinical domain since 2018. MEDSTS [7], consisting of 1068 curated sentence pairs, was used in the BioCreative/OHNLP challenge task in 2018 [8]. In 2019, over 1000 curated sentence pairs were added to the MEDSTS, renamed ClinicalSTS [9], which was used in the National Natural Language Processing Clinical Challenges (n2c2)/OHNLP. This work is a poststudy of the n2c2/OHNLP challenge.

Overall, 33 teams submitted 87 models to the n2c2/OHNLP challenge task; Pearson correlation was used as the evaluation measure, ranging from −1 (strong negative relationship) to 1 (strong positive relationship). Our National Library of Medicine and National Center for Biotechnology Information team developed an ensemble model by leveraging a range of deep learning models from 3 categories: word embedding based, sentence embedding based, and transformer based (which is described in the following sections). This model achieved a Pearson correlation of 0.8967 in the official test set, ranking second among all of the teams (*P*=.88 compared with the first rank, with a Pearson correlation of 0.9010). The top 10 best team submissions demonstrated relatively close performances with Pearson correlations of 0.85 to 0.90. According to the organizer’s overview, most of the top systems used deep learning models [9].

A Pearson correlation of approximately 0.9 suggests that the model’s predictions have a very strong correlation with gold standard annotations [10]. Such results might give the impression that deep learning models have already solved STS in the clinical domain. Nevertheless, the human-level correlation in this data set is significantly lower; for example, the agreement between 2 annotators in ClinicalSTS had a weighted Cohen *κ* of 0.6 [9], suggesting that only a moderate level of correlation was achieved by human experts [10]. Therefore, we urge caution with regard to the extremely high correlation achieved by the models (which might be potentially due to overfitting) and argue that it is critical to understand how these models perform in reality rather than further improve the performance in this data set. Therefore, in this postchallenge study, we aim to analyze the effectiveness and efficiency of 5 deep learning models in depth:

- For effectiveness, we investigate how a single deep learning model performs in this specific data set and further analyze the robustness of models in sentence pairs of different degrees of similarity.
- For efficiency, we measure the inference time taken by the deep learning models in the testing set. This is an important indicator of whether these models can be used in real-time applications, such as sentence search engines. To the best of our knowledge, few studies on STS in the biomedical and clinical domains have considered model efficiency. However, given that models have already achieved a Pearson correlation of approximately 0.90, measuring efficiency is arguably more important, as it quantifies whether these models could be used in production.

The principal findings are 2-fold. First, a single deep learning model trained directly on the official training set only (ie, without more advanced techniques, such as multitask learning and transfer learning) could already achieve a maximum Pearson correlation of 0.87; however, the training set’s robustness to sentence pairs of different similarity levels differs significantly. A particular observation is that BERT models made the most errors (a mean squared error of over 2.5) on highly similar sentence pairs (similarity no less than 4). BERT models cannot capture highly similar sentence pairs effectively when they have different negation terms or word orders. Second, although the deep learning models achieved relatively close Pearson correlations (from 0.82 to 0.87; single models), the time efficiency differed dramatically. For example, the difference in Pearson correlations of BERT and sentence embedding models was within 0.002, but the inference time of BERT models was approximately 50 times greater than that of sentence embedding models. This brings practical challenges to using BERT models in real-time applications, especially without the availability of graphics processing units (GPUs). Furthermore, although there has been a tremendous effort to make ClinicalSTS available to the community, their source corpora inevitably limit the diversity of sentence pairs and annotation inconsistencies. Thus, we call for community efforts to create more STS data sets from different perspectives to reflect the multifaceted notion of sentence relatedness; this, in turn, will further improve the generalization performance of deep learning models.

Here, we introduce popular deep learning STS methods that have been used in the biomedical and clinical domains. The methods are broadly categorized in terms of the language models applied: word embeddings, sentence embeddings, and transformers.

### Word Embedding–Based Models

Word embeddings are relatively early language models that significantly change how text is modeled. The semantic of each word is represented in a high-dimensional vector trained on large-scale corpora in an unsupervised manner. Primary word embedding methods include (1) word2vec, based on local contexts, such as using a word as input to predict its nearby words [11]; (2) Glove, based on global co-occurrence statistics [12]; and (3) fastText, which extends word2vec by adding word n-grams [13]. Many word embedding variations (eg, pretrained in the biomedical or clinical corpora, integrated with entities, and adopted retrofitting methods) are publicly available [14-16]. First, word embedding–based STS models use these embeddings to obtain vector representations of the words in sentence pairs and then use either Convolutional Neural Networks (CNNs) or recurrent neural networks to process (typically to obtain spatial or semantic patterns), followed by fully-connected layers to make predictions [16].

### Sentence Embedding–Based Models

Sentence embeddings extend word embeddings by modeling sentence-level representations. The primary methods include (1) Doc2vec, similar to word2vec, using a word as input and predicting the paragraph rather than nearby words [17]; (2) FastSent, using a sentence as input and predicting the adjacent sentences [18]; and (3) SentVec, which extends word2vec and fastText by using both words (and their n-grams) and the associated sentences as inputs for training [19]. Compared with word embedding–based models, sentence embedding–based STS models are simpler: first, they use sentence embeddings to obtain sentence vectors and then use fully-connected layers for predictions [20].

### Transformer-Based Models

Transformers are recent language models that revolutionize text representation methods. Using a self-attention mechanism, this model can capture long-range dependencies [21]. Transformer-based language models, such as BERT [22] and GPT [23], have replaced recurrent neural networks for many text-based applications. To date, many transformers pretrained in the general or biomedical and clinical domains are publicly available [24-27]. Similar to sentence embedding–based models, transformer-based STS models directly use transformers to obtain sentence representations and then use fully-connected layers for predictions [22].

## Methods

### Sentence Similarity Models

#### Overview

**Figure 1:**
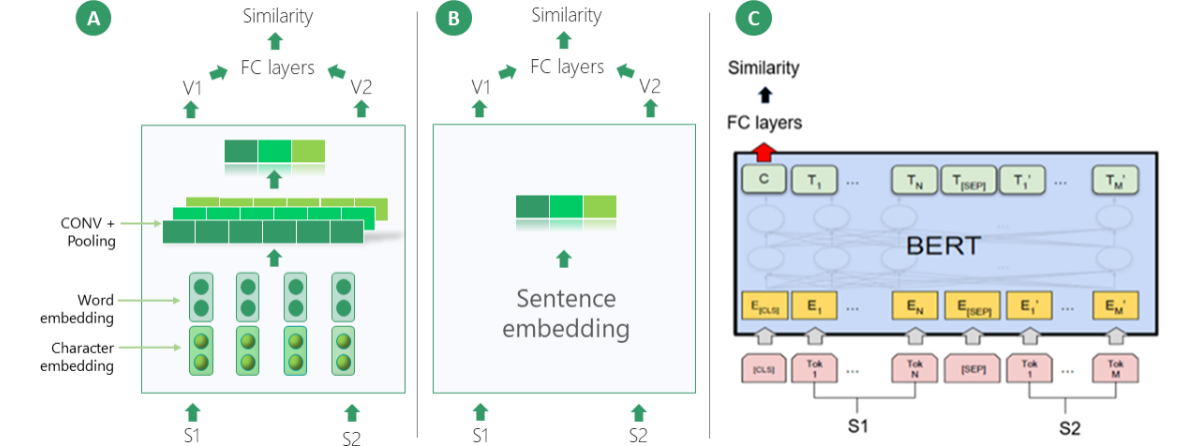
Model architecture overview. (A), (B), and (C) demonstrate the architecture of the Convolutional Neural Network (CNN), BioSentVec, and Bidirectional Encoder Representations from Transformers models, respectively. Details are provided in the Methods section. BERT: Bidirectional Encoder Representations from Transformers; CONV: convolutional layer; FC: fully-connected layer.

Five deep learning STS models from the 3 categories above were benchmarked: the Convolutional Neural Network (CNN) model [28] (from the word embedding–based category), the sentence embedding model, using BioSentVec [29] (from the sentence embedding–based category), and transformer models (from the transformer-based category), using BioBERT [24], BlueBERT [25], and ClinicalBERT [26]. We chose these models because they achieved top-ranked performance in STS-based tasks [5,8,9]. The general architecture is shown in Figure 1, and the descriptions are as follows.

#### Word Embedding–Based Model (CNN Model)

We adapted the CNN model from a study by Shao [28] a top-ranked system in SemEval-2017 Task 1. The CNN model transforms the input sentence pair into vectors and learns the similarities between the corresponding vectors. The backbone is a Siamese neural network, whereby the model weights are shared when processing the 2 input sentences. The model consists of 3 layers (shown in Figure 1A). The first embedding layer consists of word and character embeddings. It is used to transform the raw text into a 2D semantic vector space. In this study, we evaluated several word embeddings. We found that word embeddings pretrained in the biomedical and clinical domains did not have additional advantages in this specific data set. This observation is consistent with the previous word embedding evaluation using the same source data set [15]. Therefore, we used Glove pretrained in the general domain for the following experiments. The second layer consists of convolutional and max-pooling layers to extract special information from the embeddings. Therefore, the 2D semantic vector space is transformed into a 1D vector to represent the semantics of a sentence. The third layer provides a calculation of the absolute difference and dot product between the vectors of the 2 sentences. This is followed by the fully-connected layers to produce the final similarity prediction.

#### Sentence Embedding Model (BioSentVec Model)

We used the model from [29], which achieved the highest performance on MEDSTS for the post–BioCreative/OHNLP challenge task [20]. The model structure is similar to the CNN model above, as shown in Figure 1B. The primary difference is that this model uses BioSentVec to directly produce the sentence vectors. Therefore, there are no convolutional or pooling layers.

#### Transformer-Based Model (BioBERT, BlueBERT, and ClinicalBERT Models)

This model structure is illustrated in Figure 1C. First, the sentences were concatenated as one input (as recommended by the authors of BERT [22]), followed by a BERT module and fully-connected layers. We benchmarked 3 different BERT modules: (1) BioBERT [24], pretrained on PubMed abstracts and PubMed Central full-text articles; (2) BlueBERT [25], pretrained on PubMed abstracts and Medical Information Mart for Intensive Care-III clinical notes; and (3) ClinicalBERT [26], pretrained on clinical notes using the weights from BioBERT.

#### Additional Machine Learning Baseline Model (Random Forest)

Although the top-performing submissions used deep learning–based models [30], it is also critical to compare with traditional machine learning–based models to better understand the effectiveness and efficiency of deep learning–based models. Therefore, we evaluated the performance of a classic machine learning model as an additional baseline. Specifically, we adapted the random forest model, which achieved the best performance out of 13 submissions in the 2018 BioCreative/OHNLP challenge task [20,30]. This model uses manually engineered features in 5 dimensions to capture sentence similarity: token-based, character-based, sequence-based, semantic-based, and entity-based. We performed feature selection based on the performance of the validation set and ultimately selected 13 features.

### Data Set, Evaluation Metric, and Hyperparameter Tuning

The details of the data set are presented in the data description studies [7,9]. In short, the data set consists of 2054 sentence pairs, with the similarity annotated on a scale of 0 to 5: (1) 0, if the 2 sentences are entirely dissimilar; (2) 1, if the 2 sentences are dissimilar but have the same topic; (3) 2, if the 2 sentences are not equivalent but share some details; (4) 3, if the 2 sentences are roughly equivalent but some important information is different; (5) 4, if the 2 sentences are mostly equivalent and only minor details differ; and (6) 5, if the 2 sentences are semantically equivalent [7]. The data set was annotated by 2 medical experts, with a weighted Cohen *κ* of 0.60 as the interannotator agreement measure [9].

The training and testing sets were officially released by the task organizers and consisted of 1642 and 429 sentence pairs, respectively. We randomly sampled approximately 20% of the sentence pairs (329 pairs) from the training set as the validation set. The Pearson correlation coefficient was used as the official evaluation metric.

Given that the models have different architectures and hyperparameters, we performed hyperparameter tuning for the CNN, BioSentVec, and BERT models separately, rather than using the same values. The values of the hyperparameters are listed in Table 1.

**Table 1:** Hyperparameters of the sentence similarity models. Common hyperparameters are shared among all of the models. In contrast, model-specific hyperparameters are only for specific models.

Hyperparameters			CNN^a^		BioSentVec		BERT^b^ variation
**Common hyperparameters**							
	FC^c^ layers	128		512, 256, 128, 32		128, 32	
	Dropout	0.5		0.5		0.5	
	Optimizer	Adam		SGD^d^		AdamWarmup	
	Learning rate	1e-3		5e-3		2e-5	
	Batch size	64		16		32	
**Specific hyperparameters**							
	Maximum length	170		N/A^e^		128	
	Conv^f^	1800		N/A		N/A	
	Pooling	Maximum		N/A		Maximum	

^a^CNN: Convolutional Neural Network.

^b^BERT: Bidirectional Encoder Representations from Transformers.

^c^FC: fully-connected.

^d^SGD: stochastic gradient descent

^e^N/A: not applicable.

^f^Conv: convolutional layers.

### Evaluation Methods

We measured the Pearson correlation (for effectiveness) and the running time in seconds (for efficiency) on the testing set. To compare the 5 models quantitatively, we repeated the experiments 10 times on the same training, validation, and testing sets and reported the results of Wilcoxon rank-sum test on the average Pearson correlation and running time at 95% CI. We chose the same evaluation metric and statistical test as the task organizers for consistency [9]. We further evaluated the Spearman correlation, R², and mean square error as additional metrics for effectiveness.

In practice, the running time can be significantly affected by the computing environment rather than the model architecture. For instance, GPUs could significantly boost the inference time; however, many sentence search servers (especially research tools) may not have GPUs available. Different multi-processing methods may have an impact on the running time as well. For a fair comparison, we used a single processor on the central processing unit for model inference on the testing set and tracked the running time accordingly.

## Results

### Effectiveness and Efficiency Results

Table 2 presents the effectiveness and efficiency results. All 5 deep learning models had reasonable and very close effectiveness results for this data set. The difference between the average Pearson correlation was within 3%. The BioSentVec model achieved the highest Pearson correlation (0.8497), followed by BioBERT (0.8481; *P*=.74). The deep learning models had approximately 15% higher Pearson correlation than the baseline random forest model. In addition, the results demonstrate that a single deep learning model can achieve a maximum Pearson correlation score of 0.87. We further developed a model by averaging the predictions of the 4 best models. The ensemble model further improved the score by close to 0.90. This observation is consistent with our submission results. Table 3 provides additional effectiveness measures. BioSentVec consistently showed the highest performance in 3 out of 4 metrics, followed by BioBERT.

**Table 2:** Effectiveness and efficiency results for the official test set. The models are ranked by the mean effectiveness results in descending order. The *P* value of the Wilcoxon rank-sum test at a 95% CI is shown for each model compared with the model with the highest effectiveness or efficiency results. The results of the ensemble model also are provided; however, this study focuses on single models in terms of, for example, their robustness to sentence pairs of different similarity levels and their inference time for production purposes.

Model			Effectiveness (Pearson correlation)							Efficiency (seconds)				
			Values, mean (SD)		*P* value		Maximum effectiveness		Values, mean (SD)			*P* value		Lowest efficiency
**Five benchmarking models**														
	BioSentVec	0.8497 (0.0099)		N/A^a^		0.8654		1.48 (0.23)			N/A		1.96	
	BioBERT	0.8481 (0.0122)		.74		0.8698		85.05 (4.93)			<.001		95.66	
	ClinicalBERT	0.8442 (0.0161)		.39		0.8677		85.20 (4.74)			<.001		95.21	
	BlueBERT	0.8320 (0.0232)		.02		0.8613		84.81 (1.63)			<.001		88.22	
	CNN^b^	0.8224 (0.0043)		<.001		0.8307		4.35 (0.27)			<.001		4.97	
**Additional machine learning baseline model**														
	Random forest	0.6848 (0.0022)		N/A		N/A		0.03 (0.00)			.99		0.03	
**Ensembled model**														
	Ensemble model	0.8782		N/A		0.8940		N/A			N/A		N/A	

^a^N/A: not applicable.

^b^CNN: Convolutional Neural Network.

**Table 3:** Additional effectiveness results of individual models. The models are ranked by the Pearson correlation coefficient in descending order.

Model			Values, mean (SD)						
			Pearson correlation		Spearman correlation		R²^a^		MSE^b^
**Five benchmarking models**									
	BioSentVec	0.8497 (0.0099)		0.7708 (0.0073)		0.6705 (0.0325)		0.8709 (0.0434)	
	BioBERT	0.8481 (0.0122)		0.7951 (0.0100)		0.6636 (0.0275)		0.8803 (0.0362)	
	ClinicalBERT	0.8442 (0.0161)		0.8066 (0.0149)		0.6357 (0.0391)		0.9155 (0.0502)	
	BlueBERT	0.8320 (0.0232)		0.7701 (0.0244)		0.6520 (0.0544)		0.8935 (0.0670)	
	CNN^c^	0.8224 (0.0043)		0.7674 (0.0087)		0.6136 (0.0436)		0.9428 (0.0519)	
**Additional machine learning baseline model**									
	Random forest	0.6848 (0.0022)		0.6572 (0.0027)		0.4154 (0.0025)		1.1614 (0.0025)	

^a^R^2^: coefficient of determination.

^b^MSE: mean square error.

^c^CNN: Convolutional Neural Network.

In contrast to the effectiveness results, the efficiency results differed dramatically among the models. As shown in Table 1, it took about 1.5 seconds, on average, for the BioSentVec model to predict the similarities of 429 sentence pairs in the testing set; the counterpart of the CNN model took about 4.5 seconds, on average. In contrast, all BERT models require more than 80 seconds, on average, for inference.

### Error Analysis

**Figure 2:**
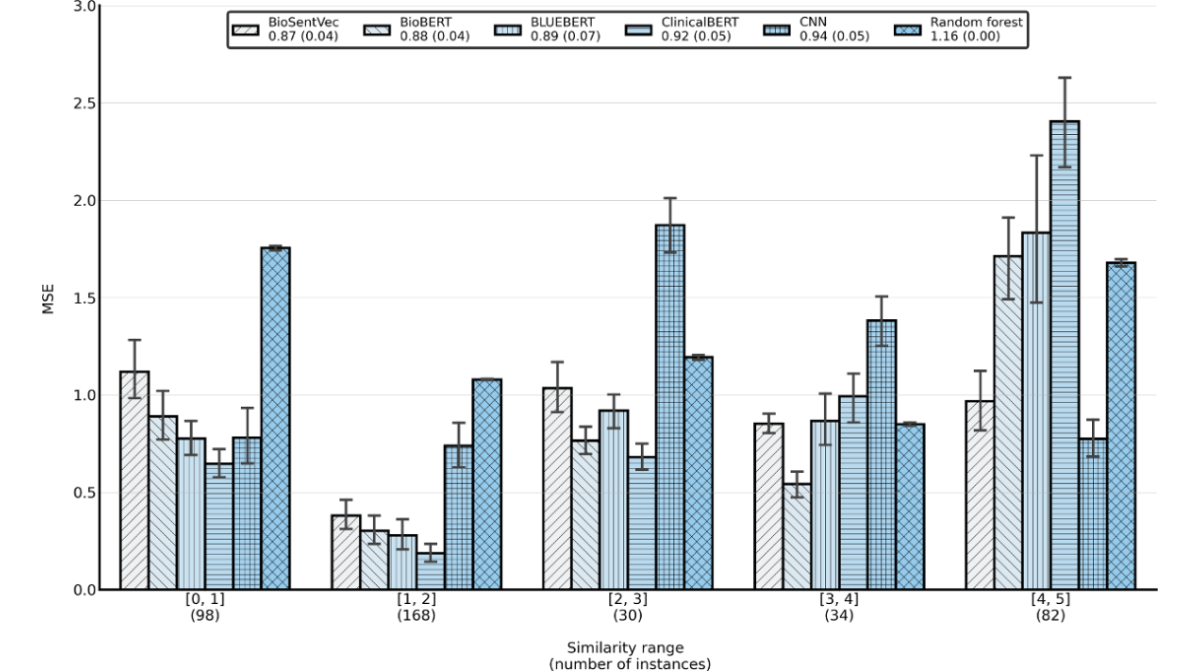
Mean squared error (MSE) of the models for each similarity range. Each category shows the number of sentence pairs and associated MSE of the models. The overall MSE (median, SD) are also provided in the legend. CNN: Convolutional Neural Network.

We further analyzed the common errors made by the models. Figure 2 shows the quantitative evaluations. We categorized the sentences into 5 groups based on the annotation guidelines and measured the MSE between the gold standard and predictions. Note that we did not use Pearson correlations as they are heavily influenced by the limited number of instances in small categories [20]. MSE is thus used as an alternative metric, which has also been used as a loss function for many deep learning models for regression-based applications.

Figure 2 shows 2 primary observations. First, the random forest model had the highest MSE for the pairs with similarity scores between 0 and 1; the error rate was almost twice that of the deep learning models. In contrast, the MSEs of the random forest in other similarity categories were much smaller. This suggests that the random forest model may not effectively identify sentence pairs of low similarity. We manually examined the sentence pairs of low similarity and provided representative examples where the random forest model had a larger MSE than the other models, along with the predictions of BioBERT and BioSentVec for comparison (Table 4). The errors shared consistent patterns where (1) the sentence structure was similar (eg, both started with “The patient...”), (2) the pairs shared many common or similar words (eg, case 4 shares “examined and normal”), and (3) the semantics of the pairs were rather different. In such cases, the random forest model failed to capture the semantics at the sentence level. In addition, cases 1-3 had the gold standard annotation score of 0, whereas the similar case 5 had the counterpart of 1. One may argue that the drugs in case 5 are rather different, and the procedure was independent and could have a score of 0; alternatively, given the score of case 5, cases 1-3 could arguably have the same score as well because they were all related to patient status (similarly, both BioSentVec and BioBERT provided consistent scores on these cases). This is also consistent with the findings of the task organizers [9], which demonstrated that annotating the sentence similarity is a challenging task as relatedness is context-dependent.

**Table 4:** Qualitative examples with a relatively large mean squared error for the random forest model for sentence pair scores from 0.0 to 1.0.

Case	Sentence pairs	Gold standard	Random forest	BioBERT	BioSentVec
1	The patient tolerated the procedure well and was transferred to the recovery room in stable condition. The patient was transferred to the patient appointment coordinator for an appointment to be scheduled within the timeframe advised.	0.0	2.5	0.5	1.2
2	Patient to call to schedule additional treatment sessions as needed otherwise patient dismissed from therapy. Patient tolerated session without adverse reactions to therapy.	0.0	3.4	1.4	1.4
3	Patient was agreeable to speaking with social work. Patient was able to teach back concepts discussed.	0.0	2.0	1.7	1.7
4	Left upper extremity: Inspection, palpation examined and normal. Abdomen: Liver and spleen, bowel sounds examined and normal.	0.5	2.4	2.1	1.1
5	glucosamine capsule 1 capsule by mouth one time daily. Claritin tablet 1 tablet by mouth one time daily.	1.0	2.6	1.7	1.5

Second, all the deep learning models, except the CNN model, showed reasonable performance for the pairs with similarity scores between 1 and 4. The MSE was mainly within 1, suggesting that the predictions were likely in the same category as the gold standard. However, the BERT models had a much higher MSE for the pairs with scores from 4 to 5. For example, ClinicalBERT had an MSE of over 2.5, whereas the counterparts of both CNN and BioSentVec were lower than 1. Similarly, the variance of BERT models on sentence pairs with similarity scores from 4 to 5 was also larger than that of the other models. Table 5 shows the representative sentence pairs for which ClinicalBERT had a larger MSE than the other models, along with the predictions of BioBERT and BioSentVec for comparison. The examples indicated that ClinicalBERT could not capture highly similar sentence pairs when there are different negation terms (eg, case 1) or when the word order is switched (eg, case 2) as compared with BioBERT and BioSentVec. Similarly, interannotator consistency may also have an impact on MSE. For example, sentence pairs from cases 4 and 5 arguably belong to the same category, as the pairs share the majority of information, except for minor differences.

**Table 5:** Qualitative examples with a relatively large mean squared error for Bidirectional Encoder Representations from Transformers models for sentence pair scores from 4.0 to 5.0.

Case	Sentence pairs	Gold standard	ClinicalBERT	BioBERT	BioSentVec
1	Heart: S1/S2 regular rate and rhythm, without murmurs, gallops, or rubs Heart: S1, S2, regular rate and rhythm, no abnormal heart sounds or murmur	5.0	2.5	3.4	3.9
2	He denies chest pain or shortness of breath He denies shortness of breath or chest pain	5.0	2.3	3.3	3.9
3	This patient benefits from skilled occupational and/or physical therapy to improve participation in daily occupations Medical necessity: the patient would benefit from skilled physical therapy interventions to be able to return to work and engage in self-care activities	4.0	2.4	2.2	2.5
4	All questions were answered to the parent’s satisfaction All questions were answered and consent was given to proceed	4.0	2.8	2.6	3.7
5	The patient understands and is happy with the plan The patient verbalized understanding and wishes to proceed	5.0	3.0	2.9	3.6

## Discussion

### Principal Findings

This study has 2 primary findings. First, the effectiveness of deep learning models on this data set is high (all 5 models have a Pearson correlation of over 0.8, which is approximately 15% higher than that of the traditional machine learning model) and relatively close (the Pearson correlation difference is within 0.03 among the models), but their efficiency is significantly different. BERT models are, on average, 20-50 times slower than the CNN and BioSentVec models, respectively.

The dramatically different efficiency results lead to the concern of using STS models in real-world applications in the biomedical and clinical domains. To demonstrate this, we further quantified the number of sentence pairs that could be computed in real-time based on the sentence search pipeline in LitSense [2]. LitSense is a web server for searching for relevant sentences from approximately 30 million PubMed abstracts and approximately 3 million PubMed Central full-text articles. To find relevant sentences for a query, it uses the standard BM25 to retrieve top candidates and then reranks the candidates using deep learning models. The rerank stage in LitSense is allocated for 300 ms based on evaluations of the developers. Using 300 ms as the threshold, BERT models can rerank only 2 pairs in real-time, whereas the CNN and BioSentVec models can rerank approximately 30 and 87 pairs, respectively. It should be noted that the results here are for demonstration purposes. In practice, as mentioned above, many factors could impact the inference time, such as GPUs and efficient multi-processing procedures. The real inference time might differ, but the difference between the models holds, as we fairly compared all of the models in the same setting. On the basis of these results, we suggest using compressed or distilled BERT models [31] for real-time applications, especially when production servers do not have available GPUs.

The second primary finding is that the random forest model made more errors in sentence pairs of low similarity (similarity scores from 0 to 1), whereas BERT models made more errors on highly similar sentence pairs (similarity scores from 4 to 5). The random forest model cannot effectively capture the sentence semantics when a sentence pair shares consistent structures and similar words but distinct topics. In contrast, ClinicalBERT had an MSE of over 2.5 for highly similar sentence pairs, especially when different negation terms or the word order is switched. As mentioned above, the results also suggest that interannotator consistency may also impact MSE, showing the difficulty of relatedness-based tasks.

### Limitations

The main limitation of this study is that the analysis was conducted using the ClinicalSTS data set alone. To the best of our knowledge, the data set is already the largest available sentence similarity data set in this domain. Other data sets, such as BOSSES, are much smaller. We believe that it is critical to developing more sentence similarity data sets from other sources in the biomedical and clinical domains, which could expand our analysis and further improve the existing methods.

Another limitation is that the ClinicalSTS data set lacked user-level evaluations. The notion of relevance is context-dependent: sentence pairs with high similarity scores predicted by the models may not necessarily be considered relevant by users [32]. Previous studies demonstrated that the top sentences ranked by the top STS models were not the most relevant to users based on manual judgment [33]. Therefore, it is critical to conduct user-level assessments to understand whether STS models can facilitate information retrieval in practice, in addition to understanding the effectiveness and efficiency measures. We consider this as future work.

### Comparison With Prior Work

Most existing studies focus on developing innovative methods to improve correlations in the testing set. Top-ranked methods are summarized in the overview papers on clinical STS challenge tasks [8,9], from traditional machine learning methods [30] to word and sentence embedding–based methods [20] and transformer-based methods [24]. Other studies further used advanced learning methods, such as representation fusion [34] and multitask learning [27]. The reported Pearson correlations range from 0.83 to 0.90, which is consistent with our study. Although it is exciting to further improve the state-of-the-art results, it is more critical to understand the effectiveness and efficiency of these models in depth, especially when the human-level correlation level is only moderate in these data sets.

Only 2 studies have compared the effectiveness of STS models in the biomedical and clinical domains [35,36]. Tawfik et al [35] compared the performance of a range of embeddings in sentence-based data sets (mostly classification-based applications, not STS) in the biomedical domain. Studies have shown that embeddings pretrained in biomedical and clinical corpora could achieve reasonable Pearson correlation scores, which is consistent with our study. However, these studies focused mainly on the Pearson correlations and did not consider model robustness or efficiency. Arguably, the latter is more critical to using STS models in practice.

### Conclusions

In this postchallenge study, we comparatively analyzed the effectiveness and efficiency of 5 deep learning models in the ClinicalSTS data set. Although these models achieved high Pearson correlation scores, their robustness varied dramatically in terms of sentence pairs at different similarity levels, and BERT models have significantly longer inference times. In addition, the models achieved Pearson correlations of approximately 0.90 in this data set, whereas the human-level agreement was only moderate. Taken together, these observations make us cautious about the further improvement of this data set and argue for a more thorough evaluation of the model-generalization capability and user-level testing. We also call for community efforts to create more STS data sets from different perspectives to reflect the multifaceted notion of sentence relatedness, which will further improve the generalization performance of deep learning models.

## Abbreviations

BERT: Bidirectional Encoder Representations from Transformers
CNN: Convolutional Neural Network
GPU: graphics processing unit
n2c2: National Natural Language Processing Clinical Challenges
OHNLP: Open Health Natural Language Processing
STS: semantic textual similarity
